# Integrating family integrated care into neonatal practice: nursing experiences and education program development—A qualitative study

**DOI:** 10.3389/fped.2026.1717431

**Published:** 2026-02-12

**Authors:** Sibel Gunduz, Colin Morgan, Emily Hoyle, Mark A. Turner

**Affiliations:** 1 Department of Women’s and Children’s Health, University of Liverpool, Liverpool, United Kingdom; 2 Department of Neonatology, Liverpool Women’s Hospital, Liverpool Women’s NHS Foundation Trust, Liverpool, United Kingdom

**Keywords:** family centred care, family integrated care, neonatal intensive care unit, neonatal nursing education, nurse perspectives, perceived education needs, qualitative research, thematic analysis

## Abstract

**Introduction:**

Family Integrated Care (FICare) is an approach that empowers parents to become primary caregivers in the Neonatal Intensive Care Unit (NICU), placing them at the centre of their baby's care. In practice, there is a diversity among nurses regarding how they view and implement FICare. An effective education and training programme must consider this diversity. Understanding both nurses' perspectives and their training needs is critical for the long-term, sustainable, and effective implementation of the FICare model. This study aims (1) to explore nurses' views and perspectives regarding the implementation of FICare in the NICU, and (2) to assess the FICare-related educational needs of NICU nurses in preparation for a curriculum update.

**Methods:**

This initial phase of an exploratory sequential mixed-methods study employed in-depth, face-to-face interviews with 23 NICU nurses, utilising a semi-structured questionnaire that included hypothetical scenarios.

**Results:**

The main themes from the inductive thematic analysis were empowering parents, supporting parents, teamwork (including support when stress is mirrored between families and nurses), FICare benefits, FICare barriers and challenges, and professionalism. Nurses emphasised the importance of individually assessing families' needs, the ripple effect of their approaches on families, and the significance of mutual interaction. They also noted that bonding and fostering family unity are the most crucial aspects of FICare. Their effects on families can last longer than the nurses' contact with them. Comprehensive education and ongoing guidance further supported effective nurse engagement in FICare. Nurses' most perceived education needs were conducting difficult conversations and providing emotional support to parents.

**Conclusion:**

We identified key facilitators, challenges, nurses' attitudes, and specific educational needs related to FICare based on nurses' experiences. These findings provide valuable guidance for improving the implementation of FICare practices in the NICU and offer essential insights to support the development of a neonatal nursing education curriculum.

## 1 Introduction

When an infant is admitted to the NICU, separation from parents can last for an extended period, with adverse effects on both the infant's development and the parents' well-being (1). Parental stress increases and may negatively impact the mother's psychological, physiological, and social well-being (2, 3). Separation and limited communication can also complicate the bonding and hinder the development of the maternal role (4). Parents may experience stress when comparing their parenting role with that of other new parents (5). Prolonged neonatal hospitalisation can further increase insecurity, as mothers may struggle to establish their role and bond with their newborn in the neonatal unit. This might also cause feelings of insignificance and being treated as mere visitors, which can be detrimental to the maternal role (6).

Over time, comprehensive models have been developed to facilitate parent-infant interaction and reduce the negative effects of separation. However, many parents continue to feel disconnected from their infants, experience anxiety, and are uncertain about their ability to care for their infants at home after discharge. Parents report feeling like visitors who are inconsequential to their infant's well-being in the NICU (7). One early example of a humane care model was implemented in Estonia in 1979, initially driven by limited healthcare resources (8, 9). In this model, parents became the primary caregivers and had unrestricted access to their infants. They gave reports during ward rounds, recorded data in medical charts, and received education from nurses. This approach empowered parents during hospitalisation and after discharge. Preterm and full-term infants cared for by their mothers showed significantly greater weight gain during the first 30 days of life compared with those cared for by nurses (8).

Building on these findings, a Canadian team developed the Family Integrated Care (FICare) model and conducted a pilot study involving 31 FIC infants and 62 matched controls. In this study, breastfeeding data were collected from enrolment until discharge. At discharge, the breastfeeding frequency in the FICare group reached 82.1%, compared with 45.5% in the control group (*p* < 0.05). This finding indicates that the FICare intervention, which lasts at least three weeks, was associated with breastfeeding frequency at discharge. Moreover, the study also showed that FICare enabled parents to gain knowledge and confidence and helped to establish positive relationships between staff and other parents (10). Thereafter, they conducted a multicenter study across Canada, Australia, and New Zealand involving 1786 preterm infants. They reported that FICare significantly increased high-frequency breastmilk feeding at discharge (≥6 feeds/day: 70% vs. 63%, *p* = 0.016) and the rate of feeding directly at the breast (33% vs. 9%, *p* < 0.0001), while also reducing parental stress and anxiety (11).

Evidence also suggests long-term benefits of FICare beyond discharge for both infants and parents. Church et al. reported that very preterm infants who received FICare showed better self-regulation and fewer dysregulation problems at 18–21 months corrected age, partly mediated through reduced parenting stress (12). Moe et al. found that Alberta FICare was associated with a lower risk of communication delay between 6 and 24 months corrected age among moderate to late preterm infants, suggesting a potential protective effect on early development (13). In addition, qualitative findings from Shafey et al. indicated that FICare improved fathers' confidence, sense of role, and overall NICU experience, with positive effects persisting after discharge (14). FICare was also associated with reduced maternal chronic physiological stress at 18 months, as measured by cumulative hair cortisol, and reductions in maternal stress mediated improvements in child internalising, externalising, and dysregulated behaviours (15).

Despite these benefits, implementation of FICare in NICUs remains inconsistent and influenced by multiple contextual and organisational factors. Nurses, who act as mentors and guides for parents, experience this shift from traditional care models in different ways. Studies show variation in healthcare professionals' readiness to share responsibility with parents, their interpretations of their evolving role, and their consistency in applying FICare principles (16, 17). Parents likewise report uneven communication and inconsistent information they receive from staff (16, 18). Family engagement is further shaped by cultural background, language, knowledge, emotional readiness, socioeconomic status, and caregiving expectations. Some families feel completely included and supported, while others, particularly those from minority or disadvantaged groups, encounter barriers such as judgment, lack of information, or limited power sharing in care decisions (16). Systematic reviews underline that parental involvement depends not only on their readiness but also on staff attitudes, communication styles, environmental conditions, and organisational culture (19). Across studies, a recurring message is that both families and health professionals need clearer, more consistent guidance (17, 20). Parents often seek more information, emotional support, and reassurance, while healthcare professionals highlight gaps in training, uncertainty about how to operationalise FICare, and difficulties managing the complex needs of families from diverse sociocultural backgrounds (11, 17, 18).

Accordingly, there is potentially a complex mix of attitudes, knowledge, and intentions that need to be addressed in an educational programme. This diversity must also be taken into consideration both in care practices and in the professional education programs to be developed for this approach. Despite the existing FICare training in the study settings, challenges remain in translating FICare principles into everyday practice. Recognising this complexity, the purpose of our integrated series of studies (work programme) is to refine and further develop the existing educational programme to reinforce professionals' positive attitudes to FICare and address diversity among families. This particular study is the first step in the work programme and aims to guide improvements in the existing FICare training curriculum by answering the following questions:

1. What are the nurses' views and perceptions of FICare among nurses with experience of FICare?
2. Which facilitators and challenges to FICare do the nurses encounter in its implementation?
3. What are the nature and diversity of attitudes, knowledge, and intentions that need to be addressed in the design and improvement of an educational programme for nurses practising FICare?

## 2 Materials and methods

**Figure 1:**
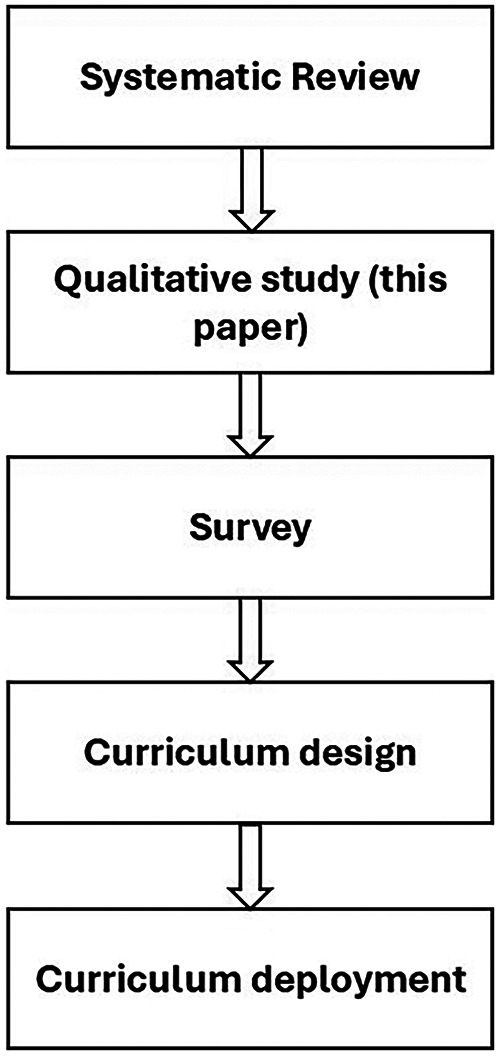
Overview of the research programme informing FICare education development.

We adopted a sequential exploratory mixed-methods approach to meet the aims of the research programme (21). This paper reports findings from the qualitative phase of this programme (first phase) (Figure 1). In this phase, the study sought to gain a detailed understanding of participants' experiences and perspectives, examine the research topic comprehensively and flexibly, and highlight participants' expressions and meanings. The themes identified in the qualitative phase formed the basis for developing the survey in the next phase (reported separately). Accordingly, in a subsequent study, the quantitative data were utilised to measure the dimensions identified in the findings of this study and to test them within a larger sample.

The research was conducted in two NICUs in Liverpool, managed by two NHS Trusts through the Liverpool Neonatal Partnership (Liverpool Women’s Hospital and Alder Hey Children's Hospital). The Partnership hosts almost 7200 births a year. Both units provide tertiary care, including pre- and post-operative surgical care, with a total of 4,500 intensive care days a year. ECMO is available on one of the sites. Nurses providing intensive care have three years of undergraduate training, one year of preceptored training in NICU, and a recognised neonatal intensive care (QIS) certificate within two years of starting on the Unit. The sampling frame includes nurses working at these hospitals. FICare is actively implemented in both NICUs. In our clinics, in line with the FICare Accreditation Criteria for the Northwest of England, >85% of nurses and medical staff should be trained in FICare. The research sample comprises nurses working at these hospitals who agreed to participate in the study. The hospitals include low-dependency, high-dependency, and intensive care units. In one hospital, parents are permitted to stay 24 h a day, with a recliner chair at the bedside. At the other hospital, each family stays in a private room with a bed available.

Pilot interviews were conducted with the first four participants to refine the interview guide. Minor revisions were made to improve question clarity, and these pilot interviews were included in the final analysis, as no substantial changes to the core questions were required.

We used maximum variation sampling, a type of purposive sampling. Nurses were selected based on their experience in neonatal nursing and the level of care they provided. The main goal of maximum variation is to include participants with different characteristics, experiences, and perspectives, ranging from typical conditions to extremes. Its basic principle is to understand a phenomenon as comprehensively as possible. Data saturation refers to the stage of analysis where no more information is generated, resulting in a recurrence of previously identified data. In our study, we reached data saturation with a total of 23 nurses from both hospitals.

Potential participants working in NICUs were asked to contact the study team if they were interested in taking part in interviews. The participant information form was sent by email. Face-to-face interviews were conducted during nurses' shifts in a quiet room of the NICU to create a comfortable environment for honest responses. The interviewer is an experienced neonatal nurse (> 15 years' clinical experience) but has not been a member of the clinical team in the participating units. The research was explained to participants using the participant information leaflet, ensuring they fully understood the data handling procedures and consent requirements. Written consent and permission for audio recording were obtained. The interviews lasted approximately 20–30 min. Identity information was kept confidential, and each participant was assigned a study code number. The audio recordings were stored in encrypted folders within secure digital environments and were only accessible to the research team. During the interviews, participants were reminded that they could withdraw at any time or omit questions as needed. Open-ended questions allowed for detailed responses, and field notes recorded both verbal and non-verbal cues to provide context (22).

A semi-structured prompt guide was developed as a data collection tool (see Supplementary File S1). The guide included scenarios to explore participants' viewpoints in a structured yet flexible manner. The guide was designed to provide semi-structured, open-ended questions, thereby enabling in-depth responses while maintaining consistency throughout interviews. A semi-structured interview ensures that each interview covers the same topic, yet allows the interviewee to interpret and associate freely (23).

Through the integration of scenarios, the interview encouraged participants to reflect on real-world situations, improving the richness and contextual relevance of their responses. This strategy was intended to capture the complex and nuanced perspectives of participants.

The data obtained from interviews were analysed using the thematic analysis method developed by Braun and Clarke (24). NVivo 14 software (Denver, CO, USA) was used in the analysis process and manage the data systematically. Themes were created inductively. After the interview, the records were transcribed verbatim. Transcription and analysis processes have been conducted primarily by the first author, SG, with support from the senior author, MAT. The data were analysed inductively in line with the six-stage thematic analysis process (24). The Consolidated Framework for Implementation Research (CFIR) was applied as an interpretive lens to organise the emergent themes (25).

Lincoln and Guba's (1985) four trustworthiness criteria—credibility, dependability, confirmability, and transferability—were employed to ensure the reliability of the data analysis (26, 27).

Credibility refers to confidence in the truthfulness of the findings. Prolonged engagement in the clinical setting, along with in-depth, face-to-face interviews with nurses at varying levels of experience and across shifts, attention to context strengthened the study's credibility. All steps were explicitly documented.

Dependability concerns the consistency of data across time and varying conditions. The research design and data collection procedures were thoroughly described. During thematic analysis, coding, theme development, and interpretation were reviewed collaboratively with MAT to ensure an external perspective. Peer debriefing sessions improved interpretive accuracy.

Confirmability addresses the extent to which the findings are shaped by participants' accounts rather than the researcher's assumptions. An audit trail was maintained, including interview transcripts, codes, analytic memos, developing themes and reflexive notes, to allow the logic of the interpretations to be traced.

Transferability refers to the applicability of findings to other settings. This was enhanced by providing descriptions of the clinical setting, participants' experience level, interview conditions, data collection procedures, and illustrative participant quotations. The study does not aim for generalisation but offers enough detail for readers to assess the relevance of the results to their own clinical environments.

The study was reported in accordance with the COREQ guidelines (see Supplementary File S2) (28).

The ethics review was conducted by the Health Research Authority (HRA) and the University of Liverpool ethics committee.

## 3 Results

Twenty-three nurses were interviewed. Participants' professional experience varied widely, ranging from less than one year to more than 30 years (Table 1). Additional sociodemographic data were not collected to protect participant confidentiality within the clinical setting.

**Table 1:** Participants’ professional Neonatal Intensive Care Unit (NICU) experience.

Experience category	*n*	%
Less than 1 year	3	13.0%
1–5 years	7	30.4%
6–10 years	9	39.1%
11–15 years	1	4.3%
16–20 years	0	0%
21–30 years	2	8.7%
More than 30 years	2	8.7%
Total	23	100%

**Figure 2:**
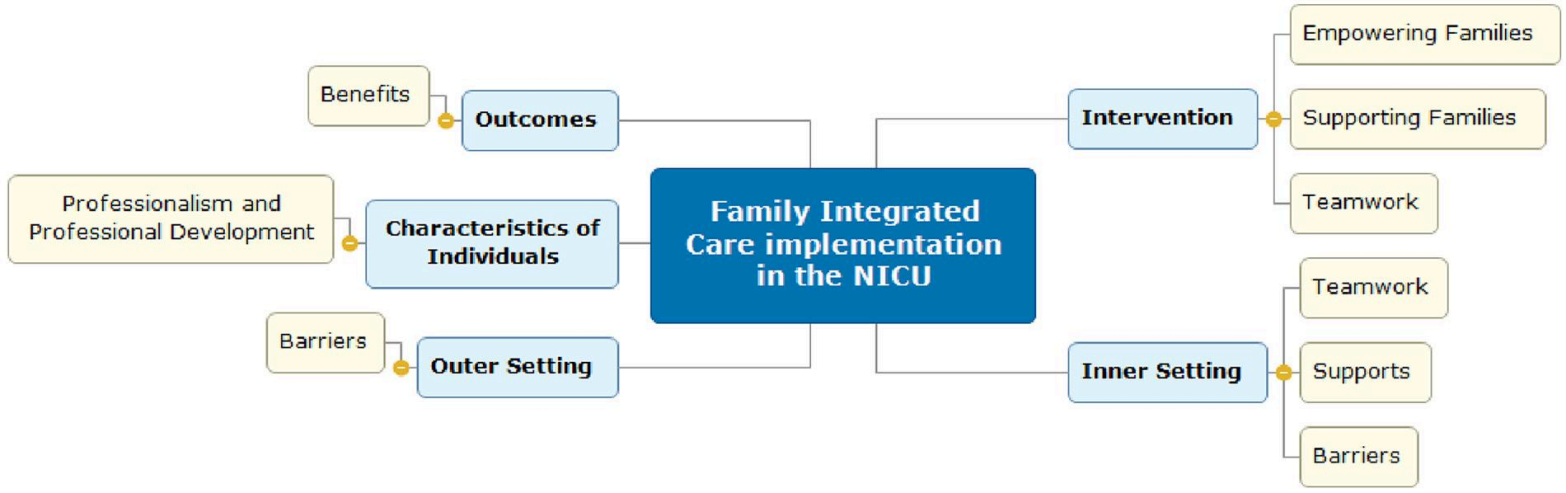
CFIR-based framework of FICare implementation.

Six themes were identified through inductive thematic analysis: (1) empowering families, (2) teamwork, (3) professionalism, (4) supporting families, (5) FICare benefits, and (6) FICare barriers and challenges, all of which are vital for the successful implementation of the FICare model (Table 2). The CFIR examines factors influencing implementation across five domains: intervention characteristics, outer setting, inner setting, characteristics of individuals, and the implementation process. Following inductive thematic analysis, the emergent themes were subsequently mapped onto CFIR domains (Figure 2). Empowering families and supporting families were mapped to intervention characteristics, reflecting core components of the FICare model. Teamwork was mapped across both intervention characteristics and inner setting domains, capturing its role as an integral element of the care model as well as its dependence on organisational communication structures and shared working practices. Professionalism was mapped to characteristics of individuals, encompassing nurses' knowledge, skills, and beliefs, and to process-related factors such as training and professional development. The FICare barriers theme spanned both inner setting factors, including organisational constraints such as staffing and time pressures, and outer setting factors related to family circumstances affecting participation. The benefits theme reflected perceived outcomes associated with FICare implementation.

**Table 2:** Themes, subthemes, definitions and illustrative quotes.

Theme	Definitions	Subtheme	Exemplary Quotes
Empowering families	The process of enabling families to gain knowledge, skills, and confidence to participate actively in their infant's care and clinical decision-making.	Family involvement	“I got them involved and made them feel like this is their baby.” (S5)
		Family education	“If they weren't happy doing it, I'd say you'll watch me, and then maybe you try the next one.” (S16)
		Empowerment in decision-making	“I think in the first few days of life, it's very hard for them to make balanced decisions.” (S20)
Teamwork	Shared goals, effective communication, and mutual respect characterise collaborative working relationships among healthcare professionals and families.	Collaboration	"I feel like the health professionals could possibly work as a team a bit better.” (S20)
		Shared responsibilities between staff and parents	“If we miss something, parents can tell us when a change has happened since yesterday.” (14)
		Communication with families	“If they're quite shut down and not showing much emotion, then sometimes it can be harder to engage with them.” (S22)
Professionalism	The ongoing development of nurses’ knowledge, skills, and competencies is essential to implement FICare effectively.	Challenges in NICU nursing	"The hard things… like death, and bereavement.” (S1)
		Fullfillment of NICU nursing	” I do enjoy seeing the happiness.” (S2)
		Perceived nurses’ roles	“It's our role to support them as much as we can in any way.” (S8)
		Perceived training needs and professional development	“I always think I'm gonna say the wrong thing.” (S10)
Supporting families	The provision of emotional, practical, and informational support to help families cope with NICU stress and adapt to their parenting role.	Identifying family support needs	"Some parents encounter financial problems, so we support them also in that way.” (S15)
		Promoting family unity and comfort	“I think playroom, then parents don't feel the need to stay home with their other children.” (S20)
FICare benefits	The positive outcomes and advantages of FICare for infants, families, and the NICU environment.	Benefits for parents and infants	“It's better for family in terms of bonding.” (S17)
		Benefits to the NICU environment	“It reduces the workload on nursing staff” (S12)
FICare barriers and challenges	The challenges and barriers that hinder the effective implementation of family-integrated care in the NICU setting	Infant and Family-Related Barriers	“It's a very unnerving area for a lot of people.” (S3)
		Systemic barriers	“Set care times can be discouraging.” (S17)

Each theme is described in detail below. Each theme is described below, emphasising implications for nurses' FICare training programmes.

### 3.1 Empowering families

Three interrelated subthemes emerged: family involvement, family education, and empowerment in decision making. These subthemes formed a progressive pathway in which active involvement fostered familiarity and confidence, structured education supported competence development, and shared decision-making positioned families as informed partners in care. Consequently, families were empowered and better prepared for discharge, with improved skills and knowledge to manage potential challenges at home.

#### 3.1.1 Family involvement

Nurses consistently described active parental involvement as the foundation of the FICare model. Involvement was framed not merely as assisting nurses but as assuming meaningful caregiving responsibilities similar to those expected at home, in line with parents' readiness and confidence. As primary caregivers, families help ensure the continuity of care for the baby and are actively engaged in the baby's care.

Nurses stated that families were encouraged to participate in care and that nurses played a key role in facilitating this involvement. As parents became more confident and willing to engage, they were described as being able to provide safe care without needing to seek permission from nurses. Nurses (identified through study code numbers) explained:

“I think they should be able to do just as much as they can with the support of nursing and medical staff. When I'm on shift, I always encourage my parents …., feel free to do …just like you would at home. If you see your baby awake and crying, you can change his nappy: you don't need to sort of ask permission or feel like you have to. It’s your baby.” (S12)

#### 3.1.2 Family education

Nurses described education as central to supporting families in maintaining continuity of care and acquiring sufficient experience during hospitalisation. They stated that systematic, gradual, and consistent teaching of basic care skills not only enhances the quality of care but also boosts families' self-confidence.

“Instead of teaching them everything, one by one, …. with the help of the nurse or with the help of dad and the next day, a little more slowly, we can teach them.” (S15)

Most nurses emphasised that education needed to be personalised and well-structured. During the education process, nurses reported that respecting and adapting to parents' learning paces is essential for successful outcomes.

“It’s been two weeks, and she hasn't done anything. She really wants to, but she’s just so stressed, and ….I said I'll do it for you, and then you can help me and watch me, and she was really happy to do that.” (S10)

Nurses also highlighted that education should not feel overwhelming and should focus on preparing families for care at home (e.g., feeding, nappy changing, handling the baby), as well as providing basic orientation to NICU equipment, in order to reduce anxiety and support continuity of care after discharge:

“Explaining what all the machines are, how to even just as simple as opening the incubator door, how to access the baby.” (S18)

#### 3.1.3 Empowering in decision-making

Nurses emphasised the importance of empowering families not only in care practices but also in decision-making processes. Parents need adequate information, emotional support, and professional guidance to reach a point where they can make decisions. Families should participate in decision-making and highlighted the significance of their involvement in ward rounds. Nurses described how FiCare increased opportunities for parents to join clinical discussions and decisions and how ward round practices had been adapted to facilitate this:

“I think this has been very good at getting parents involved in ward rounds and medical decisions, but it is not perfect and could still be improved.” (S7)

“We have changed quite a few things on the unit so that parents can now stay for the ward round. They are able to listen and get involved; they can stay in the room as long as they wear headphones.” (S4)

S20 emphasised that time should pass after admission so that families can be competent enough to make decisions. “I think in the first few days of life, it's very hard for them to make balanced decisions. I think it can be done with support.” (S20)

Participants reported that parents needed to fully understand their infant's situation before participating in decisions.

“I think it's good to give the parents power and involve them in decision-making; you want to make sure that you fully understand. Like what steps would be taken, …. and when they'll be moving into a cot and things like that, and feeding, because it's their baby.” (S22)

### 3.2 Teamwork

Teamwork emerged as a key theme, covering collaboration, communication within the team, and shared responsibilities between staff and parents.

#### 3.2.1 Collaboration

Nurses often emphasised the importance of interdisciplinary collaboration, noting that integrating expertise from various support systems—such as the breastfeeding team, the FICare team, neonatal community outreach team, psychologists, social workers, and local services—is essential for meeting the complex needs of families. “We have psychologists on the unit, who we can get to speak to psychologists if they want to.” (S16)

S20 stated that voluntary organisations could also offer additional support for families and work together with them.

“Maybe we could bring volunteers back to help care for mums while they're in the unit. I know that there are a lot of volunteer organisations that support breastfeeding; I don't know if they'd have the manpower to come in and help mums with breastfeeding and stuff.” (S20)

They also emphasised the importance of doctors and other senior nurses in ensuring families' involvement.

“I think the doctors would make a big difference to some staff, especially the least experienced staff. On ward rounds, if something they suggested to the nurse is probably more likely to be done, while if it wasn't brought up, it might not occur to some staff.” (S10)

Participants expressed concerns about the healthcare team's limited interprofessional collaboration. While acknowledging that each professional carried out their duties, some emphasised a lack of effective communication and mutual understanding between disciplines. S20 proposed organising structured opportunities, such as joint sessions, to understand better each other's roles, and referral pathways indicated a perceived need for improved team integration and more transparent collaborative processes.

“I feel like the health professionals could possibly work as a team a bit better. I feel like everybody does their own job and doesn't always communicate with other professionals.. I don't know how you get.. Maybe some more sessions where you get to meet the professionals and learn a bit more about their jobs and how to do referrals.” (S20)

#### 3.2.2 Communication within the team

Caring for a neonate in the NICU requires a team effort, involving nurses, parents, doctors, educators, administrators, and other healthcare professionals. Nurses stated that families are the most vital part of the team. “They are the most important people in the baby's care.” (S1) Open communication with families was identified as vital for care, with nurses noting that families expressing emotions constructively lead to more effective nurse-family interactions. “If they're quite shut down and not showing much emotion, then sometimes it can be harder to engage with them.” (S22) It has been emphasised that the importance of adapting to families' communication styles. In line with this, S18 explained that effective family communication requires a flexible attitude, considering each family member's preferences and emotional expressions.

“Some parents are really chatty, ask a lot of questions, and are quite happy, and you know, you can mirror that energy. However, if you've got a really anxious family, I feel like sometimes you have to tone down a little bit, speak a bit quieter, and adjust your mood that way.” (S18)

Receiving positive responses from families was identified as crucial for effective communication. These positive cues, including nonverbal indicators of satisfaction and pride, reassure nurses that they are on the right track.

“The positive feedback I get from parents when there’s a milestone..They feel quite proud of themselves. That gives me a route that helps me carry on doing the same sort of thing. They do affect me quite a lot more than I realise.” (S20)

Building on the importance of collaboration, nurses emphasised that open and regular communication within the team is essential for maintaining consistent care plans and building trust with parents. Nurses noted that consistent communication and information sharing across shifts are vital for gaining parents' trust and ensuring continuity of care.

“Someone comes in, checks and changes. Someone else comes in the next day, and it’s a different change. Especially for parents, that causes a lot of anxiety……I think that trust wavers a bit.” (S23)

Nurses emphasised the importance of providing accurate information to families. They also mentioned that there is always someone in the team available to ask about anything they do not know.

“If I'm not sure, I'll get the information about it, or I'll go get hold of one of the doctors or the ANNPs (advanced neonatal nurse practitioners), ….I don't want to wing it. I want to make sure they get the correct information.” (S19)

Nurses use comprehensive documentation to keep all team members informed about significant changes and progress in the infant and family. This practice promotes consistent care and fosters a shared understanding of each child's unique journey. Tools such as passports and achievement cards record progress in a simple, family-held format, helping parents observe milestones and celebrate the collective efforts of the care team. “We have the passports here on the unit and the achievement card things, so we can see what they can do for the baby.” (S8) Despite recognising its value, S12 stated that they are not very good at documentation. “I think probably the passports that we use, we were not very good at using them in here.” (S12)

#### 3.2.3 Shared responsibilities between staff and parents

Nurses emphasised that clearly defined roles, responsibilities, and shared goals among team members are essential for effective infant care. By spending the most time with their baby, participants recognised that families are often the first to notice changes and can play a vital role in early detection of issues. “I feel like they play an important role in recognising things because they're with their baby all the time.” (17)

However, nurses acknowledged that families could feel stressed or anxious if expected to monitor clinical issues, such as the baby's IV site. To support families without overwhelming them, nurses recommended that they retain responsibility for clinical monitoring, ensuring parents are involved in ways that are empowering but not burdensome.

“But potentially to be the person to have to make those changes, I think that’s quite a significant role to play, so on them as well, that might make it a little bit unfair, I feel like that'd be another additional pressure to add on to what they already do.” (S17)

Nurses emphasised that families should be able to do everything for their newborns that they would do at home.

“They should be able to do all the care that they would do at home with the baby that they'd be expected to go home to do with the baby. So, anything like nappies, washing, you know, bed changes, holding.” (S17)

However, they said some limits are necessary for the baby's safety. As one nurse said, “I think parents should be involved in almost everything safe for the baby.” (S1). Another nurse noted, “Making sure that the baby's safe when they do it.” (S15).

### 3.3 Professionalism

This theme explored the aspects that nurses find most enjoyable and challenging aspects of the NICU, along with their professional roles and perceived training needs.

#### 3.3.1 Most enjoyable and challenging aspects of the NICU

When asked what they enjoyed most, participants highlighted monitoring babies' good progress, their preference for looking at babies rather than adults, observing families' confidence, witnessing discharges, and sharing families' happiness, revealing a strong link between satisfaction and emotional attachment.

One participant stated, “And I think I enjoy working with the families and seeing the progression of the patients, so having them for a long time from the very premature until going home, discharged.” (S22) Another participant said, “I enjoy how resilient the babies are. I enjoy seeing them take big milestones and make leaps in their development. I find it really satisfying.” (S7)

Participants identified the main challenges as infant deaths, deterioration, unexpected bad events, workload, and fear of saying the wrong thing to families. A participant explained, “When a baby is really unwell and dealing with the parents, sometimes it is quite complicated, and you do not always know the right thing to say and or do.” (S1) “I always think I'm gonna say the wrong thing.” (S10) Another nurse shared,

“There’s a lot of sad outcomes…. especially when you're present for the conversations that aren't so positive, and you kind of anticipate poor outcomes, I think that’s probably one of the worst parts.” (S17)

#### 3.3.2 Perceived nurses' roles

Many nurses described supporting and empowering parents as a central aspect of their professional role in the NICU. This support involved educating parents, encouraging their participation in care, and ensuring that they felt confident and comfortable performing caregiving tasks. As one participant stated, “It's our role to support them as much as we can in any way.” (S8) Another nurse similarly noted, “We're there to support them and to educate them to ensure that they're comfortable doing certain tasks.” (S19)

Nurses emphasised the importance of identifying families' support needs and facilitating access to appropriate support. One participant explained:

“I think it means making sure that parents have the support they need to care for their baby. If they require additional support from us, or from family members or counsellors, this should be provided so that they can give what they can to their baby while they are in the NICU.” (S22)

In describing their professional role, nurses highlighted personal and professional attributes they considered essential to NICU nursing. Empathy and a non-judgmental approach were frequently mentioned as key characteristics underpinning their practice. One participant said, “I think I always put myself in their shoes, like this is their baby.” (S10) Another nurse noted, “We are naturally a caring profession, so I would like to think we’re all quite sympathetic towards families and understanding of their situation.” (S23)

Nurses had differing views on whether FICare changed their professional role. Some experienced nurses described continuity in their roles, while acknowledging that transferring aspects of care to parents could be a change for some colleagues. As one participant explained:

“I don’t really feel like my role has changed. Even when I first started working many years ago, I felt I was there to support families and that they were the most important part of what I do… It’s about looking at our practice in a more formal and standardised way across units. But I do speak to colleagues who find it a little bit uncomfortable to hand over what they traditionally felt was their care.” (S3)

Another experienced nurse also observed that FICare was more a change in terminology than in actual practice. “It's always been there. It hasn’t really changed my role… it just changes the name and how we promote it.” (S9)

On the other hand, less experienced nurses perceived a more apparent shift in practice following FICare implementation. “In the past, it was usually the nurse taking over the care. Now it's about changing the culture and supporting parents to take on that care, with parents at the centre of the baby's care.” (S10)

“From my point of view, it’s a very good thing that I've practised before; there are no FICare. So, it’s a new experience for me since I came here. Back where I used to work, we're treating parents as visitors.” (S14)

One participant stated: “I think it's a really good thing, because I can clearly see the difference from when I was a student to how things are now.” (S20)

Nurses also noted that the length of professional experience influenced perceptions of family involvement. As their years of experience in NICU nursing increased, most nurses reported feeling more confident in involving families in care and teamwork.

“If you're less experienced, you're less inclined to allow the parents to do more, so you need a quite firm relationship with family to know what they feel comfortable with, what they're able to do, and what you feel comfortable letting them do.” (S3)

However, some nurses reported that less experienced nurses can identify changes in care practices and apply FICare more effectively. One nurse expressed:

“I think probably nurses who have worked in the NICU for a long time might struggle with changing their practice in that way.” (S7)

Another nurse stated, “I think consistency is difficult because we've had people who have been here for such a long time, and they’re still stuck saying what they would have said a long time ago.” (S4)

Nurses recognised the need to regulate emotions to maintain professionalism during a shift. Some described consciously separating emotional situations. One nurse said, “When you walk into the room, you sort of flip a switch… you’re that person until you walk out. (S11)” Others highlighted that professionalism meant following procedures and prioritising patient and family needs regardless of modes: “We have procedures in place in hospitals, so I just follow the procedure.” (S4); “That would be unprofessional in my eyes… they’re my priority, and I do the best I can.” (S9).

Participants described relying on peer support and individual coping strategies to manage stress and continue providing care. As one nurse reflected, “We’re only human… it's just trying to lean on other staff or support as well and try and give the best care you can.” (S18).

#### 3.3.3 Perceived training needs and professional development

Nurses consistently emphasised that professional development was crucial, and FICare training helps keep their knowledge current and consistent. S10 stated, “I think education and continuity are a big thing, and it's hard to keep continuity in a big unit.” (S10)

##### 3.3.3.1 Identified priority training needs

Based on participants' responses, the following codes were identified as perceived educational needs and recommendations for sessions, each directly connected to challenges within other themes (*n* refers to the number of participants who mentioned this need):

Having difficult conversations (*n* = 3): Nurses reported anxiety around communicating with families during difficult events, deterioration, or poor prognoses. This training directly addresses the challenges described earlier and aligns with both Supporting Families (providing emotional support during difficult situations) and Empowering Families (enabling informed decision-making even under challenging circumstances). S1's statement, “you do not always know the right thing to say” exemplifies this gap. Training in difficult conversations would equip nurses to navigate these emotionally charged interactions while maintaining family involvement and trust.

Emotional support to parents (*n* = 7): This was the most common training need that nurses recognised. S9 explained this gap: “I think the most helpful would probably be psychological counselling, how to overcome parents who are very anxious or mums with postnatal depression, just because we never been taught much about it. So, it would help us to know how to help parents better who struggle.” This need fits with the Supporting Families theme, where emotional support is seen as essential, but nurses feel unprepared. Without this skill, the Barriers and Challenges theme shows that parents' emotional distress is still a major obstacle to FICare.

Teaching skills (*n* = 5): Teaching families well is key to Empowering Families, but nurses saw gaps in their teaching skills. S7 said, “I think I can probably educate them on some things. I can certainly educate them on nappies and tube feeds, but I'm still learning a lot of skills myself.” Nurses agreed that education should be personalised, gradual, and respect each family's learning pace, but they need more structured ways to teach. Better teaching skills would help nurses adapt to different family learning styles, cultures, and readiness, making families more confident and capable when they leave the hospital.

Time management (*n* = 2): Some nurses mentioned that workloads and limited time hindered full family involvement, as highlighted in the Barriers and Challenges theme. S19 said, “ So sometimes you haven't got the time to pack them with the parents, actually, you could go and do a set of cares in 15–20 min tops, and the parents can take up to an hour to do that” This highlights a struggle between duties and engaging families. Providing time management training for family integrated care could help nurses balance their duties with family education and support.

Beyond identifying specific content needs, nurses emphasised the critical importance of training continuity, standardisation, and accessibility across different experience levels.

Training for new nurses: Participants mentioned the importance of training new nurses. New nurses said they have basic education but need help putting knowledge into practice. S11 noted that when families are very involved, it can sometimes limit learning for new nurses: “I think as someone new, I feel like sometimes it can affect your own learning. And if a parent's already doing everything, you're sort of watching them sometimes.” Still, S11 recognised the long-term value, “I think for the long term.. It's really important for the parents to do and to learn.” S1 highlighted the need to support new staff, “We've got a lot of new starters on the unit at the minute.. we do FICare teaching once a year on our blockbuster training study days.. in each of the rooms there's quick folders with all FICare information.”

Ongoing Education and Booster Sessions: Nurses stressed that FICare training should be an ongoing process with regular reinforcement. S11 recommended: “Maybe education for nurses…. anything new or anything we need to be aware of….more like a booster lesson.” S1 noted the recent prominence of FICare and current implementation efforts: “This is something that's really become prominent in the past couple of years, and it's something that we're really trying to promote on the unit and get accredited for.” The nature of FICare practice requires continuous learning, as S16 observed: “I feel like we're constantly learning. There will be things that I still don't know that I need to learn, and it's constantly changing as well.”

Standardisation to Ensure Consistency: A concern emerged about inconsistent nursing practices, leading to confusion among families. S4 noted, “I think it's more down to training and teaching. So we have study days and blockbuster days, where we're all taught the same thing.. It's about training to get everybody saying consistent things.” S4 further described, “Because parents do get very confused with conflicting advice.”

Knowledge Sharing and Research Dissemination: Nurses expressed a need for a systematic approach to sharing FICare knowledge and research findings to support the development of evidence-based practice. S16 suggested: “I still think it's very new, so maybe just still carry on sharing the knowledge and the research that keeps going into it.” They also expressed their wish to closely follow nursing practices and family participation models in various countries so that they can compare and improve their own practices.

“I'd like to hear a bit more about what other countries do because sometimes you get used to what you do. It's interesting and helpful to hear about a different way of doing things that you might not consider, that might be done better, and might be challenging in practice. I think it would be nice to hear from parents, like a video recording of a parent’s experience.” (S20)

This desire for comparative learning and parent perspectives indicates that training programmes should include diverse FICare models and family stories to expand nurses' understanding and question established practices. Practice-Oriented Training Approaches: Nurses said training should go beyond theory and help with real-world challenges. S11 said: “We get a good base knowledge to sort of support parents. And it's just.. more putting it into implementing it and putting it into practice and building sort of more knowledge when you sort of face certain obstacles. You don't know what you're going to get.”

Deepening FICare Knowledge: Some nurses felt they had a basic understanding of FICare but wanted to learn more. S22 said: “I think I have a good basic understanding, and then to go deeper into FICare. I'd have to read up on it.” This shows a need for training that starts with the basics and moves to more advanced topics.

### 3.4 Supporting families

This theme focused on identifying and addressing families' support needs (including emotional support), promoting family unity, and facilitating family comfort. It highlighted the facilitators and supports that addressed the challenges and barriers described in the FICare Barriers theme, and the two themes were therefore closely interconnected. In some cases, facilitators and support required collaboration with additional resources, linking this theme to the Teamwork. However, the main emphasis remained on the facilities and practical solutions identified by participants to meet family needs.

#### 3.4.1 Identifying and addressing family support needs

Nurses indicated that when families struggle to participate in infant care, their first step is to identify the root causes and determine what specific support is needed. Families may face obstacles because of personal or environmental factors. These include emotional or psychological challenges, financial hardship, other children, or distance from the hospital. Such families may need both information and different types of assistance. One nurse said, “We can find out if they need additional support.” (S13)

Another nurse expressed, “You can have a parent who has a mild learning disability, and then you can highlight certain things that they are struggling and might need a little bit more support as well.” (S19)

This approach showed that nurses assess families' situations without judgment and initiate support to facilitate involvement. When families required further assistance, nurses coordinated additional resources to support them. As one nurse expressed, “I was probably looking at what kind of support within the community that could maybe offer.” (S17)

A primary barrier preventing some families from engaging in the care process was financial hardship. Nurses described how some parents experience economic challenges and require financial assistance. Nurses emphasised that families should not be left unsupported but should be included with appropriate resources. As a nurse noted, “Some parents encounter financial problems, so we support them also in that way.” (S15)

Additionally, housing was a significant need for families from outside. Especially during extended treatment, proximity to the hospital was crucial. In these cases, hospital-provided accommodation was offered. A nurse described this solution, “We've got our family flat over the road.” (S1)

Nurses stated that many families involved in NICU care experience intense anxiety. Coming to the hospital every day and witnessing fluctuations in their babies' health status can be stressful and emotionally draining. This shows that families need not only information but also emotional support. They mentioned that they use active, empathetic approaches to understand the causes of family anxiety. They observe families and offer guidance and support. Nurses stressed that identifying emotional needs is important for healthy parental involvement. A nurse described, “Probably sit down and talk to her and ask her why she is afraid, and try to get down to the bottom of the issue.” (S11)

Another nurse explained the extent of this emotional burden with the following words: “It must be really hard and stressful coming in daily and seeing their baby's ups and downs.” (S22)

Such observations revealed that nurses considered not only the medical but also the psychosocial dimensions. Indeed, one nurse stated, “It's not focused on the medical side of what we're doing.” (S3)

Nurses indicated that emotional support is essential for most families, but they need to enhance their skills.

“I think the most helpful would probably be psychological counselling, how to overcome parents who are very anxious or mums with postnatal depression, just because we never been taught much about it. So, it would help us to know how to help parents better who struggle.” (S9)

They also stated that having teammates or team leaders who can provide support when needed is a facilitating factor.

#### 3.4.2 Promoting family unity and comfort

Participants noted that fathers can often be overlooked and need encouragement. They also emphasised the importance of including siblings in the baby's care to help the whole family integrate. Allowing siblings to visit the NICU enabled families with other children to attend more easily and helped siblings adapt to the newborn. Playrooms for siblings minimised challenges during visits and provided enjoyable experiences for children.

“I think playroom, then parents don't feel the need to stay home with their other children. They could bring them in and keep them entertained and be here with their baby.” (S20)

Such practices enabled families to be physically present, feel comfortable, and engage meaningfully in care.

It was stated that such practices enable families to be more physically accessible, more comfortable, and enable them to participate more effectively in the care process.

Nurses identified as facilitating families' participation in their baby's care were ensuring parental comfort in the intensive care environment and providing easy access to necessary interventions. Nurses emphasised the importance of allowing sufficient individual space next to the incubator, ideally with a bed or at least a recliner chair, as well as the availability of rest rooms and education rooms. They also underlined the need for solutions that enable the participation of individuals with disabilities, the presence of staff who can provide support when required, and the provision of appropriate spaces for private conversations. Allowing families to bring personal items from home was considered to strengthen bonding with the baby and to make the NICU environment feel more familiar. Nurses further noted the value of baby diaries, baby passports, memory-making activities, and celebrating milestones.

“That's why the parents feel well with the baby, and they feel like they are not useless. They feel like they're able to do things with baby, and they feel a bit more relaxed in that environment because it is so hard to get them to relax, especially if the baby's very poor.” (S19)

### 3.5 FICare benefits

Another theme from the data is FICare benefits. Nurses highlighted the advantages of FICare for the mother, the baby, and their own work environment based on their personal experiences. This theme revealed nurses' shared views on the benefits of FICare for infants, families, and healthcare professionals.

#### 3.5.1 Benefits to parents and infants

The main thing participants shared about FICare was that it helps with parent-infant bonding and brings the family together. “It's better for family in terms of bonding.” (S17)

Nurses highlighted that FICare is linked to improved clinical outcomes for babies, such as stabilised vital signs and increased maternal milk production through skin-to-skin contact. They mentioned both emotional and physiological benefits.

“It shortens the baby’s length of stay, improves bonding, breastfeeding rates, and satisfaction. Babies are more likely to have better outcomes in the long run.” (S10)

Participants mentioned that FICare boosts parental confidence through education and participation, enabling parents to attend ward rounds and be involved in care decisions. This helps lessen anxiety and prepares families for discharge. As one nurse said, “When that baby goes home, it gives the parents the confidence and the reassurance that they know how to care for that baby” (S1).

Participants reported that FICare enhances parents' emotional well-being. Nurses observed that mothers experienced less depression when they were actively involved in their infant's care and maintained physical proximity. As S13 noted, “Some moms are very, very depressed… and if they are holding the baby, they feel more well.”

Most participants agreed that FICare enhances the emotional bond between parents and babies and enriches the overall family experience. Participants highlighted FICare as an effective educational resource, equipping parents with knowledge and practical skills and supporting the well-being of both parents and infants. This enables families to take a more active role in their child's care. This is reflected in the nurses' statements as follows: “It gives parents confidence and empowers them” (S22). “When it comes to discharging them, much more prepared.” (S20)

#### 3.5.2 Benefits to the NICU environment

FICare also transformed the care environment. Nurses noted that this approach created a more welcoming and less intimidating space for parents, shifting away from a purely clinical atmosphere and making it more comfortable.

“I think it can make everything a bit less daunting and create an environment that feels less clinical. It lets you do everything you should be able to do with your baby whenever possible.” (S17)

According to participants' opinions, it fostered a stronger relationship between staff and families. This approach was seen to bridge the gap between healthcare professionals and families.

“FICare has helped me build better relationships with parents, as it gives me more opportunities to support and reassure them about their concerns.” (S19)

FICare also improved the staff's work environment. As parents became more skilled and confident in providing care, nurses reported needing less assistance. For example, one participant (S12) explained how FICare reduces workload over time: “It reduces the workload on nursing staff.. It does significantly reduce the burden and the pressure on neonatal nursing staff.” As a result, this eased workloads and promoted shared responsibility.

Nurses regarded FICare as highly beneficial. It results in better outcomes for babies, enhances family support, improves the work environment for staff, and adopts a comprehensive approach to newborn care. They mentioned that these advantages should be communicated to families and new staff to raise awareness and encourage family involvement.

### 3.6 FICare barriers and challenges

Although the recognised benefits of FICare are acknowledged, nurses identified several barriers or challenges. The data revealed four subthemes: the infant's clinical condition and systemic challenges, communication difficulties, parental emotional distress, and parental individual factors. These barriers interacted in complex ways, reinforcing one another and making it harder for families to participate in their infants' care.

#### 3.6.1 Infant and family-related barriers

When infants were extremely premature, very sick, ventilated, or connected to multiple monitoring devices, families were often hesitatant to take part in care and feared causing harm. Some participants mentioned that less experienced staff felt uncertain about handling or moving infants in incubators. Tubes, wires, and incubators were described as creating physical and emotional barriers to parental involvement.

“Parents might not want to get their baby out. Because they're worried about these wires, maybe more junior staff. I've only been here a year, so I feel very nervous getting a ventilated baby out of the incubator. So, a lot of staff might try to avoid that.” (S20)

Nurses further described how parental fear and anxiety reduced willingness to participate in care, particularly when parents were worried about losing their baby. They mentioned that some parents delayed bonding and that it could take days or weeks for parents to feel emotionally prepared to engage caregiving activities. This emphasised the importance of psychological support services in the NICU, as emotional distress was perceived as a significant barrier to FICare when such support was limited.

“If the baby’s quite sick, especially if they're intubated, often I feel like that creates a lot of anxiety and then maybe not so willing to want to do something like a nappy change.” (S17)

Nurses highlighted that families often felt anxious and stressed, especially first-time parents and during the initial days in the NICU. Some parents hesitated to bond with their baby due to concerns that the baby might not survive. “She's frightened to bond with the baby because maybe… she's worried the baby won't survive.” (S4)

S23 described the emotional challenges that parents could experience as follows:

“It’s a very unnerving area. For a lot of people. It’s a very alien concept. It’s probably nothing anyone who’s started out in pregnancy has ever really thought about that they're gonna end up here.” (S3)

S4 mentioned that sometimes parents wish to go home due to the extended stay in the NICU.

“If you've got parents that sometimes we can get parents that have found it quite difficult and can be quite challenging, because they've been here for a very long time, and they just want to go home.” (S4)

S18 noted that parents can take a long time to overcome their fears.

“I think another barrier is when parents don't want to because they're scared, and you know, it can take a long time to break that barrier down just because they're so anxious about it.” (S18)

The NICU environment can be quite scary for parents. Nurses expressed constant alarms, many staff, emergencies, and high-tech devices created a chaotic environment for families.

“I think the NICU environment is quite wild. It’s a busy environment with a lot of people and different members of the team. So, it’s hard to keep a nice quiet environment on a day shift.” (S22)

#### 3.6.2 Systemic barriers

Nurses said that keeping healthy and sick babies together in the same room can cause emotional discomfort for families and make bonding harder. Parents may need to leave for another family's privacy during emergencies, disrupting their caregiving and emotional connection. Emergencies with other infants may also disrupt family involvement. Parents were often asked to leave the room to protect privacy, which interrupts the continuity of care and parent-infant bonding.

“The neonatal environment can be quite stressful. So obviously, it’s really difficult to reduce stress. But sometimes it can be quite sick babies in a room with quite well babies. I feel like seeing those babies, and then obviously, parents want to get out and cuddle their well baby and want to be involved with them. I've heard some feel a little bit guilty when they've seen the parent, they might not be able to get their baby out for a cuddle. I'd say that’s probably one of the main difficulties because you don't want them to feel it’s already a stressful environment. So, but there is no easy solution.” (S17)

Despite the 24-hour service and family visiting permissions, there are times when individual reasons may prevent them from coming to the unit. “Obviously, we are a 24-hour service. So, we allow parents to come 24 h a day, but parents might not be able to come.” (S2)

Some nurses felt that their workload was increasing. S19 said that it is easier for the nurse to provide care than to educate and assist the family. Nurses expressed:

“So sometimes you haven't got the time to pack them with the parents, actually, you could go and do a set of cares in 15–20 min tops, and the parents can take up to an hour to do that” (S19)

“Time, sometimes you don't always have the time to go through things with parents, or sometimes it’s just easier and quicker if you were to do things yourself.” (S1)

Set care times and rigid routines can affect a family's engagement. For example, if a parent misses a care time, they may feel excluded: “Set care times can be discouraging, especially if parents can’t participate in special moments like changing nappies.” (S17)

Nurses noted that families come from diverse cultural and personal backgrounds, each bringing different expectations and beliefs about care. These differences sometimes pose challenges for staff trying to foster consistent engagement: “Families are all different and come from varied backgrounds. They have different beliefs about care and expectations from nurses.” (S19)

## 4 Discussion

This study explored nurses' perceptions of FICare in the NICU and revealed a multifaceted understanding of FICare implementation. The findings extend beyond individual attitudes, illustrating how the interaction between intervention design, professional roles, organisational context, and family-related factors shapes nurses' experiences. Six interrelated themes emerged: empowering families, teamwork, professionalism, supporting families, FICare benefits, and FICare barriers. When viewed through the CFIR framework, these findings suggest that the implementation of FICare depends not only on nurses' willingness to involve families but also on the alignment of core intervention components, individual readiness, organisational capacity, and external contextual influences.

The findings also highlighted nurses' perceived educational needs. The results can inform the refinement and strengthening of the nurse training programme aligned with FICare principles.

### 4.1 Empowering families

The concept of empowerment refers to the processes that enable individuals and groups to gain control over their own lives (29). In the context of FICare, this can be defined as the process of providing patients and their families with access to the information, skills, and resources necessary to participate in healthcare and decisions (30). Empowerment is recognised as a fundamental part of FICare (20).

Within this model, neonatal staff have a dual role: providing care to the newborn while also supporting mothers and fathers in developing their parenting role. To develop these special skills, some educational programmes for nurses have been provided within FICare (31). FICare actively involves and integrates families into their baby's care process (32). Ensuring active family involvement was a particular focus of nurses in our study, consistent with FICare's core philosophy, which positions parents as essential care partners rather than observers (20, 33). Recent systematic reviews confirmed that while healthcare professionals recognise FICare's value, shifting the practice paradigm remains challenging (17). Parents can feel unfamiliar and scared when entering the NICU for the first time. Parents expressed difficulty in seeking help from staff, even though staff are eager to assist them (31). Similarly, Dijkstra and colleagues reported that intensive care unit healthcare providers' attitudes toward family participation ranged from positive to hesitant, reflecting both positive experiences and perceived barriers (34).

The findings of our study are consistent with previous studies emphasising that families' information needs should be met with regular education programs. However, it has been stated that nurses may have difficulty in time management while providing this education, which reveals the importance of structured education programs for families (35).

The process of involving families and providing education is essential in the process of FICare and both concepts are inseparable parts of each other. Our findings show that nurses see family education as an important empowerment tool also supports the study by Brett et al., who showed that informing parents about the NICU environment, treatment process, and infant care increased parents' sense of self-efficacy and reduced their anxiety (36). Recent evidence from Turkey confirmed that FICare's structured educational component enhances parental readiness for discharge and home care while positively affecting infant outcomes, including weight gain and breastfeeding continuation (37).

Our findings identified that some families miss ward rounds due to life circumstances (e.g., work, having other children, distance) and, as a result, feel excluded from decision-making, highlighting an equity issue. This extends Sigurdson et al.'s documentation of how socioeconomically vulnerable families face greater barriers to NICU presence and participation (16). In the Broom's study, all parents expressed satisfaction with the opportunity to participate in ward rounds, but only one parent initially found the process somewhat intimidating (31). Healthcare professionals observed that parents could advocate for their infants during visits, consistent with findings that parents wish to discuss medical topics with neonatologists to build strong relationships regularly (38). Franck et al. found no overall differences in weight gain between infants receiving mFICare and those receiving FCC; however, when parents were more actively involved in key elements of the programme, such as participating in rounds and receiving parent mentoring, infants showed greater weight gain (39). Although our study did not directly assess infant outcomes, such results suggest the potential impact of family involvement.

Contribution to training: Based on the findings, it is recommended that nurses' training programmes incorporate strategies to develop personalised approaches for each family to ensure active family involvement in infant care, encourage participation in clinical rounds, and support the continuity of this involvement. Furthermore, the process of identifying and regularly assessing family needs should be approached within an iterative framework, and family empowerment should be systematically promoted.

### 4.2 Teamwork

Effective communication and teamwork are essential parts of care in NICUs. Wreesmann et al. emphasised that communication should be tailored to parents' individual needs (38). Each parent needs personal information, making communication key to supporting them (40). The Guided Family-Centred Care (GFCC) intervention provides parents with structured support to express their feelings and foster deeper communication with nurses. In contrast, communication in standard care tends to be more superficial and disorganised (41). It is recommended that doctors and nurses receive training in communication skills to support better and encourage parental involvement (41, 42).

Communication extends beyond sharing information; it is vital for involving parents in the decision-making process. Parents wish to participate in non-medical, low-risk decisions (38). Nurses have also mentioned that involving parents in these processes is essential for developing their parenting roles and taking responsibility. However, some parents are unsure how to participate. Thus, communication must be viewed as facilitating active parental engagement.

Families need positive relationships and negotiation with nurses to enhance their roles and be involved in care (43), with dissatisfaction and exclusion occurring when nurses fail to support their parenting roles. Our study showed that nurses always remember that families in stressful environments, such as the NICU, feel stressed and are separated from their babies.

Family-integrated care encourages parental involvement and promotes effective communication between parents and healthcare professionals. Effective communication provides reassurance and appropriate support to families for shared management of the newborn's care (44). As O'Brien et al. state, FICare promotes collaboration and supports parenting roles (10). In NICUs, stronger and more cooperative relationships form between healthcare professionals and families, which is viewed as essential for boosting overall job satisfaction (33).

Our finding that nurses adapt their communication style to individual family preferences and needs is consistent with Turner et al.'s observation that nurses adjust their approach, sometimes being more talkative and engaging, and at other times keeping a quieter approach (40). Communication is the area with the most individual variability and the need for standardised training. Fostering effective relationships among healthcare team members and between teams and families is crucial to the implementation of family-centred care (17).

Nurses in our study sometimes feel uncertain about their communication, especially when delivering difficult news, communicating during challenging situations, or supporting families in mourning. In one study, some nurses avoided infants and families due to communication difficulties, emotional burden after infant loss, or the belief that care interventions harmed the infant (42). Nurses encounter more communication challenges in family-centred care (FCC), often dealing with parents' anger or sadness, and they express a need for support in managing such crises (45). Still, FCC training for nurses can improve nurse-parent collaboration and communication skills (46).

Nurses recognised that teamwork is vital but acknowledged that it does not always reach its full potential. Both nursing and multidisciplinary teams contribute, but consistent communication is essential (10, 33). Families emerged as the most important members of the team. Nurses emphasised the importance of families' ongoing presence beside the baby, noting that parents who spend long periods with their infant are often the first to notice small but significant changes in the baby's condition. This finding resonates with Kowalski et al., who reported that parents identified nurses as the key source of information about their baby's condition and as the ones who communicated important changes (47). However, the frequent transfer of babies between intensive care and special care units, together with staff changes across shifts, often interrupts the continuity of information given to parents, which can make the role of families' close observations even more significant (40).

Contribution to training: Nursing education should foster teamwork awareness, emphasise its importance in delivering FICare, and nurture collaborative skills. Findings highlight the need for training nurses to handle challenging communication situations, especially emotionally difficult ones. We recommend a structured training programme for difficult conversations, including breaking bad news through real-life scenarios. FICare education for nurses needs to include support for all nurses who will educate families, including elements of “train the trainer”. Nurses need models for different approaches to families, depending on family needs, with both structured theoretical foundations and practice-oriented application through simulation.

### 4.3 Professionalism

In our study, the nurses expressed that they deeply enjoy their profession and find it rewarding. This aligns with Turner et al. (2014), who reported that nurses generally consider their work fulfilling despite certain challenges (40). Nurses enjoy observing the positive progress of babies and teaching families, and the results are comparable (40).

Nurses expressed that they dislike situations that deteriorate. Infant loss and end-of-life care cause emotional burden and moral distress (42, 48). Helplessness and profound grief related to death can result in emotional effects such as chronic fatigue and irritability (48). Moral distress stems from moral constraints or conflicts, hierarchy, or communication problems in end-of-life care, impacting both nurse and patient or family outcomes (42). These studies highlight the importance of organisational interventions to support nurses facing these challenges.

The FICare model advances healthcare professionals from their traditional role of direct caregiving to a position where they guide and empower parents (49). This requires redefining practical skills and developing cognitive and emotional abilities, such as communication, trust-building, and empathy (33). We suggest that while nurses demonstrated comprehensive knowledge of FICare, they also expressed a clear need for ongoing education and support in emotional and counselling skills (40).

During this period of change, a key source of support for nurses is their colleagues. Several participants mentioned relying on team leaders or colleagues for support when dealing with parents in emotional distress. However, many also noted a lack of formalised training in their hospitals and occasionally sought external opportunities to develop essential skills (40). Supporting nurses' personal development and providing training opportunities are said to affect job satisfaction (50).

Contribution to training: The findings are significant in highlighting the importance of FICare training for nurses. Our study revealed that nurses have comprehensive knowledge about FICare. In our study, nurses stated that they were most satisfied with caring for the family and baby, and that situations such as the baby's deterioration were the most challenging aspects of NICU. Nurses need to be given permission to make trade-offs between FICare and other aspects of care when there are pressing nursing and medical issues and when workload is high. Examples of appropriate trade-offs may be a helpful element of training.

### 4.4 Supporting families

Our findings that families' individual needs should be determined first are consistent with previous literature (40, 43, 51). Nurses indicated that fathers were frequently overlooked and pointed out the need to encourage and involve fathers in care. Similar findings were reported in studies with fathers. Fathers said that they received the least emotional support from nurses (51–53).

NICU design and the physical environment directly influence parental comfort and nurse-parent interaction. Single rooms or accommodations for families enhance privacy and caregiving participation; these setups foster parent-infant closeness and reduce emotional strain (54). The need for a comfortable space that offers all essential amenities for families' comfort and remains discreet, much like a hospital, supports parental presence and participation in care, consistent with factors influencing the implementation of family-centred care in the NICU (45).

Families often experience emotional stress and therefore require emotional support and this can be reduced through nursing support (55). Although nurses described providing emotional support to families as rewarding, but they also report it as challenging (40). When emotional support is lacking in the nurse-parent relationship, parents' feelings of loneliness increase; whereas supportive communication and the perception of parental competence enhance trust and attachment (56).

Emotional support is important for parents and health professionals. Nurses' ability to manage their own emotions also facilitates better interaction with the families of critically ill infants (42). However, the neonatal nurse's role in providing emotional support is complex, requiring ongoing staff training and support while reducing physical and staffing obstacles (40). Nevertheless, training focused on managing relationships with parents and assisting them during challenging times has been reported as insufficient (40).

Contribution to training: In our study, nurses emphasised that identifying the specific needs of each family underpins successful FICare, including the evolution of family needs and capabilities over time. FICare training can include an introduction to relevant skills and examples of family needs and how those needs inform the support offered to individual families. Accommodation and financial support were provided to families, facilitating the implementation of family participation. Nurses need a working understanding of arrangements on their unit for providing this support to families. Providing emotional support to families and ensuring their comfort is important. The provision of FICare requires patience on the part of the nurses as they adapt to and enable the development of the family's ability to meet the needs of their sick baby; relevant skills training and examples of success should be included in training.

### 4.5 FICare benefits

Nurses mentioned that regularly coming to the NICU and educating families allowed families to become familiar with all the processes of their babies, which provided continuity in the care of the baby and allowed families to be more confident at discharge. Families developing a sense of ownership in the baby's care process strengthens the parent-child bond and helps families see themselves as part of the care. These findings align with established evidence demonstrating the benefits of FICare for mothers and infants (10, 11, 32, 49).

Contribution to training: It is thought that the benefits of FICare should be included both in the education program of nurses and in the education given to families by nurses. Nurses should be introduced to disagreements between nurses and families and offered a range of approaches to disagreement that they can deploy depending on circumstances and personal preferences. Education should include ways to monitor family participation in care and decision-making, and some strategies that can be used to promote these important elements of FICare.

### 4.6 FICare barriers and challenges

Our findings show that barriers to FICare come from several sources, including the infant's health, the NICU environment, hospital routines, and parents' emotional stress. Nurses explained that when babies are extremely premature or need a lot of medical support, parents often worry about hurting their child and hesitate to take part in hands-on care. The presence of incubators, wires, and alarms can make parents feel both physically and emotionally distant from their baby. As seen in earlier studies, these high-acuity settings can unintentionally make parents think that only medical staff should handle the baby, which can lower parents' confidence and involvement (57).

Emotional distress was a significant barrier. Nurses noticed that many parents arrive at NICU already feeling anxious, overwhelmed, or afraid to bond with their baby in case something goes wrong. Other studies have also found that stress, anxiety, and depression are common for parents in the NICU and can lead to them being less involved in their baby's care. In our study, parents' anxiety increased when they got mixed or unclear information, or when they saw other babies get worse or pass away in shared rooms. Research showed that when families find information confusing or inconsistent, they feel more stressed and have a harder time communicating with staff, which can damage trust and involvement (57–59). This supportive approach helps families access information more easily and participate actively in the process (38).

Organisational issues and heavy workloads also made it harder to implement FICare. Nurses said that, because of time limits, set care schedules, and increased workload, they sometimes performed care tasks themselves rather than involving parents, even though they wanted families to take part. Other studies have found that insufficient staff and strict routines can keep parents from being there at important times and make it harder for staff to guide families as events unfold. Our data also show that families with financial difficulties, long travel distances, or other caregiving responsibilities face additional challenges in regularly reaching the NICU, which adds to existing inequalities in FICare. These results show that system-wide solutions, such as more flexible care times, help with accommodation, and a set time for family teaching, are needed to remove these barriers, rather than placing all the responsibility on parents or nurses (40, 42, 57).

Contribution to training: Identifying and removing barriers to FICare is crucial for ensuring its long-term applicability in the NICU. Identifying these barriers plays a crucial role in developing solution-focused strategies in the design of nurse training programs. Nurses should be educated about the diverse manifestations of stress in families and how to promote stress reduction. Examples of successful mediation between parents and physicians can be offered. Many nurses have these skills, but structured exposure to these skills is likely to promote effective FICare.

## 5 Limitations

While this study conducted comprehensive and in-depth thematic analyses and achieved data saturation, some limitations should be considered. The study was conducted in only two different hospitals in the United Kingdom. The long-standing implementation of the FICare model in these two institutions and the similar practices of the teams led to the findings being shaped by a specific institutional and cultural context. These results may differ, particularly in neonatal intensive care units in different countries where FICare practices are not yet widespread or where institutional support at the policy level is lacking. For example, the similarity of both vision and team functioning between the two units in the study meant that inter-institutional differences were not clearly reflected in the interviews.

Cultural diversity also presents some limitations. The study was conducted in two UK NICUs with relatively homogeneous nursing populations, and few participants had experience working in culturally diverse settings or where FICare was not routinely implemented. This may have limited insights into cross-cultural communication experiences and adaptation strategies for diverse family populations. Therefore, inferences about how communication needs between parents and healthcare professionals may vary across cultural contexts should be supported by studies in more diverse settings.

A limitation of this study is that the education needs were based solely on nurses' perspectives and did not include the views of other multidisciplinary team members. Future studies are recommended to incorporate training needs assessments from all health staff to support the development of a fully multidisciplinary education model. Another limitation of this study is the lack of socio-demographic information about participants.

Although the interviewer and primary analyst is an experienced neonatal nurse, she was not a member of the care team in either unit. The dual role as both nurse and researcher may have influenced participant responses through social desirability bias. However, this was mitigated by emphasising confidentiality, using a semi-structured guide, independent coding, and regular peer debriefing with other researchers.

Member checking was not conducted due to challenges in re-accessing participants after analysis. However, alternative validation strategies were employed to ensure trustworthiness align with Lincoln and Guba's (1985) criteria (26): pilot interviews with the first four participants to refine the interview guide; independent coding by multiple researchers; peer debriefing throughout analysis; thick description with extensive verbatim quotes; and maintenance of an audit trail (60). Interviewer bias may have led to an overemphasis on nursing-related aspects of care, but it is mitigated by independent coding and peer debriefing with other researchers.

While the findings are valid in a specific context (tertiary care in the UK), further research conducted in different NICU settings is important for transferability.

## 6 Implications for training program design

Based on the findings, a strong training program should include the elements of the CFIR framework for FICare that nurses identified, that is:

(1) cover the four main skill areas (having difficult conversations, psychological support to families, teaching skills, and time management); (2) offer regular booster sessions; (3) standardise practices to avoid confusing families; (4) include international and parent perspectives; (5) support nurses at all career stages; (6) focus on practical, scenario-based learning; and (7) provide easy-to-access resources for learning on the job. This approach would help nurses empower families, offer better support, communicate effectively within teams, overcome barriers, and fully realise the benefits of family-integrated care. Nurses differ in their experience and personal approach so that the training needs to start from, and recognise, that diversity. Families have different values, experiences, needs, and preferences, so the training needs to prepare nurses for the diversity between and within families. Supporting families to experience neonatal care as constructively as possible requires nurses to be knowledgeable about diverse experiences as well as being authentic to their own identities. In addition to a description of FICare, didactic teaching including topics identified in the interviews will be useful to nurses such as range of family responses to neonatal care; examples of trade-offs between the needs of care and elements of FICare; unit-specific arrangements for families; stress and its manifestations; benefits of FICare; approaches to mediation. Other elements of training would benefit from structured experiential learning, such as identifying family needs, empowering families, communication skills (using ongoing dialogue, feedback, and affirmation), and communicating difficult news. It will be important to promote a spirit of curiosity about families and their experiences among nurses contributing to FICare, so that they can identify and adapt to the situations they encounter. Multiple teaching methods will be needed, including information sharing, reflection, and the initial application of knowledge, skills, and behaviours in a safe environment (such as group work or role-play).

Training should include all nurses and medical staff in the NICU and be aimed at everyone. The education programme requires joint training sessions involving all healthcare professionals working in the NICU.

## 7 Conclusion

This study explored three dimensions of FICare implementation: nurses' perspectives, facilitators and challenges, and educational needs. Nurses see FICare as family empowerment through education, support, and teamwork, emphasising the vital importance of individualised support tailored to each family's unique needs and cultural background. Key facilitators include professional development and supportive environment; challenges include time constraints, staffing, and barriers limiting family participation. Educational programmes must comprise both structured and practical training, covering communication, emotional support, difficult conversations, teaching skills, time management, and integrating theoretical knowledge with hands-on simulation and bedside coaching. Sustainable FICare requires parallel investment in nurse education and organisational resources. Future research should examine diverse settings and family perspectives.

## Data Availability

The datasets presented in this article are not readily available because they contain sensitive information and could compromise participant confidentiality. Requests to access the datasets should be directed to sibel.Gunduz@liverpool.ac.uk.
