# Supplementary material for: Integrating family integrated care into neonatal practice: nursing experiences and education program development—A qualitative study
Source: Front Pediatr. 2026 Feb 12;14:1717431. doi: 10.3389/fped.2026.1717431 (PMC12935867; doi:10.3389/fped.2026.1717431)
Supplement: Supplementary file 1 [file Datasheet1.docx]

**Supplementary File 1**

**Prompt guide**

**Introduction**

My name is Sibel Gündüz. I am a neonatal nurse doing a PhD with Mark Turner and Colin Morgan at the University of Liverpool. Thank you for participating in this research. The purpose of this study is to understand how nurses and other staff work with families. This meeting is expected to take 20 or 30 minutes.

**The ‘real-world’ scenarios were built from the researcher’s experience and the literature and do not reflect any specific babies or families. Accordingly, the scenarios do not include any personal data that could identify real people.**

**Section one: General Questions**

What is your profession and role

How long have you been a health practitioner?

How long have you been doing neonatal work?

What do you enjoy the most about neonatal work?

What do you not enjoy?

**Section two: real-life scenarios**

[Note. This is the range of scenarios that will be used in the study. Each participant will not discuss all the scenarios. The interviewer will select the scenarios according to the state of the analysis and the content of the interview as it unfolds]

I would like to share some scenarios with you so that I can learn more about how you work with families.

**Scenarios**

1. Families where circumstances restrict time spent on the unit

*Mrs. Mary Jones is 30 years old. Her son, James, is two weeks old and was born at 25 weeks gestation because of pre-eclampsia.*

*Mary comes to the neonatal unit three times a week at lunchtime.*

*Mary has three other children aged 2, 3, and 5 years. Mary’s husband, Jimmy, works as a lorry driver and is away a lot of the time. Mary and Jimmy come from Scotland and their parents live in Scotland. Mary comes when her other children are in nursery or school. She cannot attend the ward because she has to take other kids to school.*

1. What do you think the parents could be feeling? (e.g. as a prompt if needed). They can feel guilty and feel not like a good mother)

2. What do you think about this scenario in clinical practice?

3. What do you do in this scenario?

1. Families worried about their roles, not know nurse’s expectations, lack of communication with health practitioners

*When Lily and William Smith come you invite them to spend time with their baby. You ask Lily how she feels about performing care for her baby. Lily says that sometimes she gets involved, and other times less so. Lily and William have to work during the day. They can come in the evening. They told you that they know what to do, that every nurse has different expectations, that they are afraid of doing something that will harm their baby. For example, some nurses said that they could feed her baby herself, while others said that they should not feed her baby alone. Father said that he does not want to feed their baby with a tube. He said it is a temporary situation and they do not need to learn it.*

1. What do you think about this scenario?
2. What do you do in this scenario?
3. How do you communicate with family?
4. What are your expectations from the family?
5. Is there a situation where you think the family should not contribute to FiCare? What?
6. What do you pay attention to when communicating with this family?
7. What can you do to avoid inconsistencies between nurses?
8. Single parents

*Ann Williams does not have a partner and she is a university student. She comes to the ward round twice a week. There is no one to support her and she does not want to come to the hospital. The baby was born at 27 weeks. He is ready to be discharged after a short period of oxygen therapy. Since the mother is 18 years old and does not have social support, the mother needs to stay in the hospital for a while and learn how to care for the baby.*

1. What do you think about this scenario?
2. How would you approach the mother in this scenario?
3. What would you do to get this mother involved in care?
4. That families have first baby (or inexperienced) are afraid and cannot do anything for caring

*A. Sarah and Tom Davies have been married for 7 years. When Mia was born at 27 weeks, Sarah suffered from depression and couldn't even come to see the baby. Sarah was only looking at photos of Mia. However, Mia was 1 month old and Sarah and Tom were asked to come to the hospital to spend time with Mia. Sarah did not come to care for their baby. When you asked why, she said she wanted to participate but didn't know what to do and was afraid.*

1. What do you think about this scenario?

2. How would you approach this family?

3. What kind of training would you plan to include in family-integrated care?

1. Suitable and willing parents

*Liz and Mike Hughes have been married for 20 years. They are in their 40s. They want to come to hospital. They have another child, she is 7 years. She would love to see the baby. The baby was born 24 weeks. The baby is going home on home oxygen. They prepared one room of the house completely for the baby. Liz and Mike need to learn to aspirate at home and feed baby with OG. The mother is willing to learn but learns slowly. Health practitioners are concerned about the development of an infection as the baby stays in the hospital longer.*

1. What do you think about this scenario?

2. How would you approach this family?

3. What is the role of the parents?

4. What do you pay attention to when educating the family?

1. Disabled families or had chronic illness.

*Ameena Roberts is 37 and has a hearing disability. She wants to care for her baby. However, she thinks that she will have difficulties in the hospital because she has a hearing disability or that she will not be able to take good care of her baby enough.*

1. What do you think about this scenario?

2. How would you approach this family?

3. What do you think about this mother caring for her baby?

4. What kind of environmental precautions should be made for such disabled families?

5. What are the problems this mother will experience while taking care of her baby?

6. What kind of measures can be taken for parents with diseases?

1. Parents with language problems, immigrant people’s problems

*Ahmet and Nuran Hamdan are refugees from Syria. Ahmet is 21 years old and Nuran is 18 years old. Their baby was born at 32 weeks. The mother speaks no English and the father knows little. Since the father is working, he can only come to ward round twice a week. It is difficult to communicate between Nuran and the nurses during the day. Communication with an interpreter is provided during the training periods. Other than that, it is explained with body language when it is necessary to communicate for a short time. However, they feel shy speaking in public.*

1. What do you think about this scenario?

2. How would you approach this family?

3. What other problems might be encountered?

4. Do you support the mother's participation in the care of the baby?

1. Issues of short staffing and other environmental problems

*Theo Roberts was born at 25 weeks and the mother did not want to come at first. Because the father was working, he could not attend the care. Now Theo is 1 month old and mother Ashley wants to participate in the baby's care. Ashley is 35 years old. She has 2 more babies (5, 8 years old) and her own mother takes care of them. Other babies were born on time, Ashley is afraid to touch her baby and thinks it will hurt her. Although she has attended the trainings, she is calling you in all care. You were helpful in the beginning, but realized that you were not making any progress.*

1. What do you think about this scenario?

2. How would you approach this family?

3 Will you continue to support this mother's involvement in the care of the baby?

4 What do you think is the way for your mother to overcome her fears?

1. Substance misuse within the family

*Penelope and Harry both stated that they were substance misusers and the mother continued to use cocaine during pregnancy. Penelope is 17 and Harry is 18 years old. Luna was born at 29 weeks and is receiving ncpap therapy. When Penelope and Harry were asked to participate in the care of Luna, both came to the hospital, but they did not participate in the care of the baby. They spent their time in the break room made for families and did not participate in education for them. The grandparents said that the children were irresponsible and that they wanted to take care of the baby themselves and wanted to participate in the care.*

1. What do you think about this scenario?

2 How would you approach this family?

3. What approach do you take to solve problems?

4 What do you think about the grandmother's participation in the care?

5 What precautions do you take before the baby is discharged?

1. Nurses with health practitioners challenges (demographic characteristics, occupational burnout and personal characteristics)

1 What is the role of the nurse in cases where the mother participates in the care of the baby?

2. What do you think about the role change?

3. Does your approach to families change when you feel good about yourself?

4. Do you experience fluctuations in your emotions during your shift, does this affect your quality of care?

5. What are the conditions that most affect your quality of care during the shift process?

*At the end of the interview ask these questions:*

Section three: FiCare perception

1. How would you describe FICare? What is it?
2. What do you think about FICare? What FiCare mean to you?
3. What are the facilitators of FiCare?
4. What are the barriers/challenges to FiCare?
5. What are your experiences implementing FiCare?

Section four: roles of parents

1. Do you think families should be able to change their oxygen level or check IV site?
2. Do you think families should stay while intubation, LP, or CPR is done?
3. What do you think families should be able to do?

Section five: role changing and communication with parent-staff and staff-staff

1. Could you tell me what you think about communication and working with families? What are the challenges?
2. What do you think about consistency with other health practitioners?
3. What do you think about your role changing?
4. Could you tell me what you experienced confliction with other staff?

Section six: lack of knowledge and training needs

1. Do you believe you have the necessary skills to educate families?
2. Do you get a sense of confidence while mentoring?
3. Could you tell me what topics would you like to include in the curriculum about FiCare?

Section seven: improvement FiCare

1. What can we improve about FiCare?

Section eight: an impressive experience

1. Could you please tell us about a story your FiCare experience?
